# A Case of Distal Nail Embedding Successfully Treated With a Slit-Tape Strap Taping Method

**DOI:** 10.7759/cureus.79018

**Published:** 2025-02-14

**Authors:** Takashi Seo, Chihiro Shiiya, Hideyuki Kosumi, Ken Muramatsu, Hideyuki Ujiie

**Affiliations:** 1 Dermatology, Hokkaido University Hospital, Sapporo, JPN

**Keywords:** distal nail embedding, ingrown nail, onychogryphosis, slit-tape strap, taping

## Abstract

Ingrown nails are a common condition, typically affecting the great toe, and are classified into lateral ingrown nails, retronychia, and distal nail embedding (DNE). DNE occurs when the nail plate's distal edge embeds into the nail fold, often following onycholysis or nail avulsion, with hypertrophy of the hyponychium obstructing nail growth. This condition causes nail discoloration, thickening, and pain, requiring treatment to restore proper nail growth. While surgical and conservative treatments are available, conservative approaches such as taping are more accessible but can be hindered by sweat or exudate. The slit-tape strap taping method, originally designed for pincer and lateral ingrown nails, provides superior adherence and longitudinal tension. We report a successful case of this method applied to a 22-year-old woman with DNE, resulting in full resolution of the condition and improvement in nail thickening after 17 months. This case highlights the slit-tape strap taping method as an easy-to-apply, noninvasive, and effective treatment for DNE.

## Introduction

Ingrown nails are a common condition, typically affecting the great toe. They are classified into three main types: lateral ingrown nails, retronychia, and distal nail embedding (DNE). DNE occurs when the distal edge of the nail plate becomes embedded in the distal nail fold, often following onycholysis or nail avulsion, where the elevated distal nail fold obstructs nail growth. The condition is believed to be caused by hypertrophy of the hyponychium, which blocks nail elongation, leading to nail discoloration, onycholysis, and nail thickening [1,2]. Consequently, as the nail plate invaginates into the distal nail fold, pain can occur. Surgical or conservative treatments are required to release the obstruction to nail growth caused by the hyponychium [1-4]. However, surgical treatments are extremely painful, and their cure rates remain unknown. Taping is a simple, cost-effective conservative treatment; however, tapes can easily come off due to sweat or exudate from the granulation tissue, and there are no reports of improved outcomes for DNE with this method. The slit-tape strap method, which adheres the tape to the skin around the entire circumference of the nail, has been shown to remain intact even in the presence of sweat or exudate [5]. This was originally designed for pincer nails and lateral ingrown nails. Given that the tension is applied longitudinally, it is also thought to be applicable to DNE, though no reports exist. Here, we report a case of DNE successfully treated with the slit-tape strap taping method.

## Case presentation

**Figure 1:**
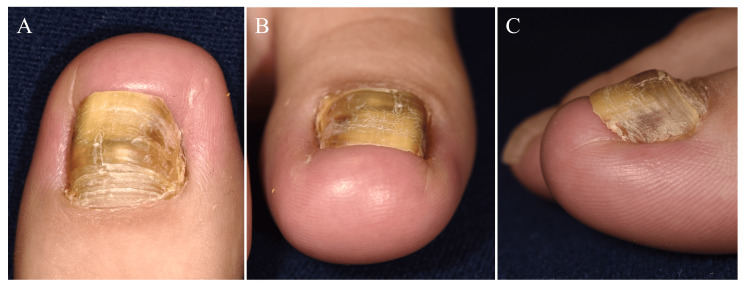
Clinical presentation. Distal nail embedding in the right great toe. Onychogryphosis is also seen.

A 22-year-old woman visited our department complaining of pain in her right toe. One year earlier, she had developed a subungual hematoma in her right great toenail after a long walk in heeled shoes. She had been experiencing pain for two months prior to her visit but had not received any treatment. This was followed by onycholysis and nail thickening, which caused the distal edge of the nail to become painfully embedded in the tip of the great toe (Figure 1A-1C).

**Figure 2:**
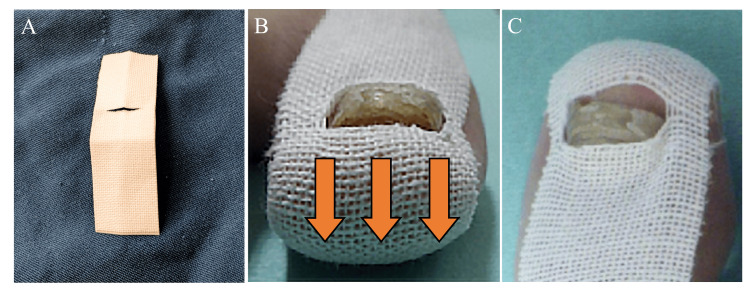
How to apply the slit-tape strap taping procedure. (A) First, an 8-cm-long strip of 2.5-cm-wide elastic tape, Elastic Adhesive Bandage “Silkytex” type 3 (Cat# 11892, Alcare, Japan), is cut and folded in half longitudinally. At one-third the length of the tape strap from the short edge, a slit is cut to the width of the nail. (B, C) After gently trimming her distal nail, the nail tip is set in the center of the slit with one side of the elastic tape oriented toward the dorsal side of the toe. One side of the tape is attached to the dorsal side of the toe, while the other side of the tape strap is attached to the plantar side of the toe, generating tension to draw the nail fold skin toward the plantar side (orange arrows).

She had no sports background, was a pharmacy student, and often wore heeled shoes. Findings of direct fungal examination and fungal culture of the nail clippings were negative. She was diagnosed with DNE, with coexisting onychogryphosis. We trimmed the distal nail plate at the point of invagination into the distal nail fold and began the slit-tape strap taping (Figure 2A-2C).

**Figure 3:**
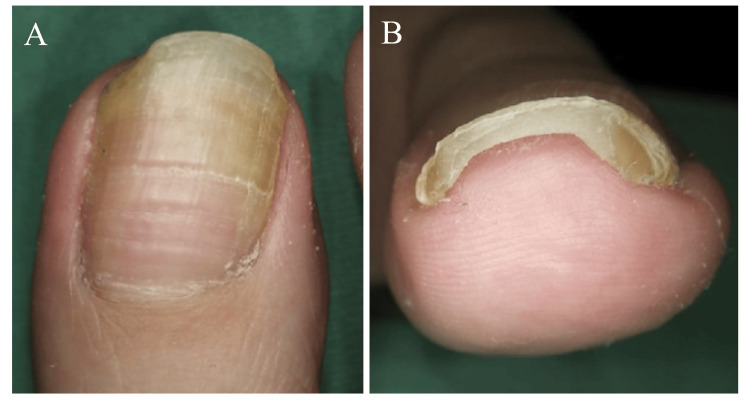
At 10 months of strip-tape strap taping. The distal nail embedding has improved, but nail thickening persisted.

After visiting our clinic, she avoided wearing heeled shoes as much as possible and returned one month later to confirm the proper slit-tape strap taping technique. Thereafter, follow-up visits were scheduled every 2-3 months. By 10 months, the nail plate had elongated past the distal nail fold, and the DNE had fully resolved, although the nail thickening persisted (Figure 3A-3B).

**Figure 4:**
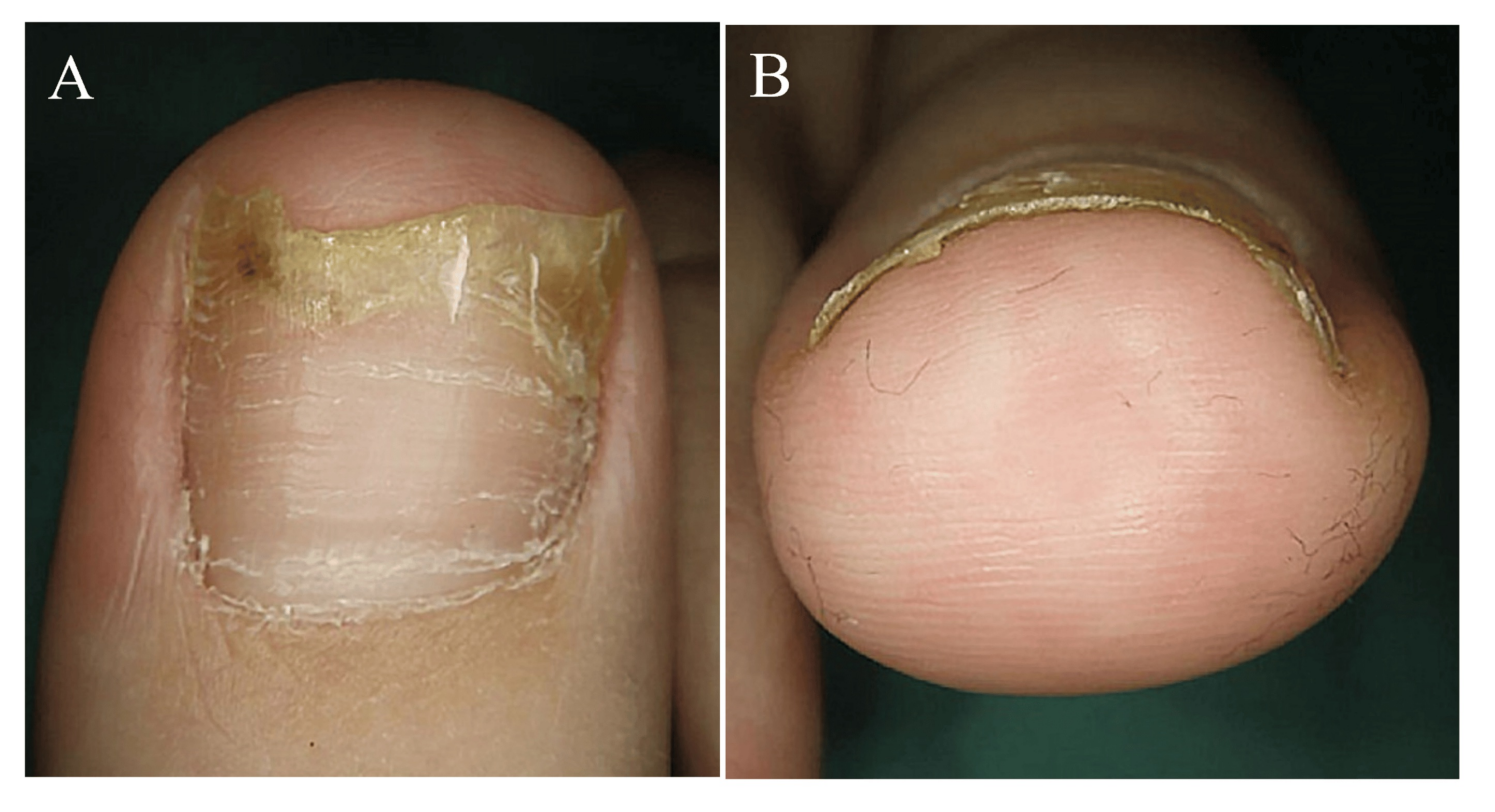
At 17 months of treatment. The onycholysis and nail thickening have resolved.

After 17 months, the nail thickening had improved (Figure 4A-4B).

## Discussion

Various methods for treating DNE have been reported [1,2,6]. However, there is no standardized approach, and different methods have been used either alone or in various combinations [1-4]. Surgical procedures can reduce the elevated distal nail fold but are highly invasive [2,3]. Moreover, even these methods do not guarantee complete restoration. As a result, less invasive treatments with more consistent success rates are highly sought after [2,6]. Conservative treatments, such as the gutter method and cotton packing, are available [2,3], but they are difficult for patients to manage independently. In contrast, taping methods are more accessible for patients to apply and manage effectively by themselves at home. Among these, the slit-tape strap taping technique is easier to apply, more stable, and less prone to peeling than other conventional taping methods [5]. It is worth noting that not only did the slit-tape strap taping method successfully treat the embedded nail, but it also improved onycholysis and nail thickening, effectively resolving the patient's onychogryphosis. 

## Conclusions

We presented a case of DNE successfully treated with the slit-tape strap taping method. Overall, the results suggest that the slit-tape strap taping method may be an easy-to-apply, noninvasive, and highly effective conservative treatment option for DNE.
